# Decreased progranulin levels in patients and rats with subarachnoid hemorrhage: a potential role in inhibiting inflammation by suppressing neutrophil recruitment

**DOI:** 10.1186/s12974-015-0415-4

**Published:** 2015-11-02

**Authors:** Chenhui Zhou, Guangbin Xie, Chunxi Wang, Zihuan Zhang, Qiang Chen, Li Zhang, Lingyun Wu, Yongxiang Wei, Hui Ding, Chunhua Hang, Mengliang Zhou, Jixin Shi

**Affiliations:** Department of Neurosurgery, Jinling Hospital, Medical School of Nanjing University, 210002, No. 305 Zhongshan East Road, Nanjing, Jiangsu Province China; Department of Neurosurgery, School of Medicine, Southern Medical University (Guangzhou), Jinling Hospital, Nanjing, Jiangsu Province China; Department of Neurosurgery, Jinling Hospital, School of Medicine, Second Military Medical University, Shanghai, China

**Keywords:** Progranulin, Subarachnoid hemorrhage, Early brain injury, Inflammation, Neutrophils

## Abstract

**Background:**

Subarachnoid hemorrhage (SAH) is a devastating neurological injury with high morbidity and mortality that is mainly caused by early brain injury (EBI). Progranulin (PGRN) is known to be involved in various biological functions, such as anti-inflammation and tissue repair. This study aimed to investigate the change of PGRN in the brain after SAH and its role on EBI.

**Methods:**

The levels of PGRN, myeloperoxidase (MPO), interleukin1β (IL-1β), and tumor necrosis factor-α (TNF-α) were detected in the cerebrospinal fluid (CSF) from SAH patients by enzyme-linked immunosorbent assay (ELISA). In addition, PGRN levels were also detected in the cerebral cortex after experimental SAH in rats by western blotting and immunohistochemistry (IHC). Recombinant human PGRN (r-PGRN) or an equal volume of phosphate-buffered saline (PBS) was administrated at 30 min after SAH. All rats were subsequently sacrificed at 24 h after SAH. Neurological score and brain water content were assessed. For mechanistic studies, the changes of MPO, matrix metalloproteinase-9 (MMP-9), zonula occludens 1 (ZO-1), Bcl-2, and cleaved caspase-3 were examined by western blotting and the levels of pro-inflammatory cytokines (IL-1β and TNF-α) were determined by ELISA. In addition, neuronal apoptosis and blood brain barrier (BBB) permeability were examined.

**Results:**

The levels of PGRN significantly decreased, and the levels of MPO, IL-1β, and TNF-α were markedly elevated in the CSF from SAH patients. In rats, PGRN levels in the brain also decreased after SAH. Administration of r-PGRN decreased brain water content and improved neurological scores at 24 h after SAH. These changes were associated with marked reductions in MPO, MMP-9, and proinflammation cytokine levels, as well as increased levels of Bcl-2 and ZO-1. In addition, neuronal apoptosis and BBB permeability were alleviated by r-PGRN.

**Conclusions:**

These results indicate that the levels of PGRN decreased after SAH and that r-PGRN alleviates EBI after SAH possibly via inhibition of neutrophil recruitment, providing a new target for the treatment of SAH.

## Background

Subarachnoid hemorrhage (SAH) is a life-threatening cerebrovascular disease that is mainly caused by ruptured aneurysms and has a morbidity and mortality rate higher than 50 % [1]. Recent studies have demonstrated that early brain injury (EBI), and not cerebral vasospasm (CVS), is the most important cause of the high morbidity and mortality. EBI is the immediate injury to the brain within 72 h after SAH [2] and strongly determines the prognosis of SAH patients [3]. Several physiological derangements occur during EBI, including increasing intracranial pressure, decreasing cerebral blood flow, and global cerebral ischemia [4]. These immediate events initiate secondary injuries, such as inflammation, blood brain barrier (BBB) disruption, cell death, and oxidative cascades [5]. Numerous findings have highlighted that inflammatory reactions are the major contributor to EBI [6–8]. Neutrophils play an important role in inflammatory reactions. Neutrophils, which are induced by inflammation, migrate into brain issue, exaggerate the inflammatory reaction, reduce cerebral blood flow, and aggravate brain injury [9]. In addition, neutrophils contribute to BBB disruption, brain edema, and neural cell injury by releasing proteases, cytokines, and chemokines [10, 11].

Progranulin (PGRN) is a 593 amino acid, cysteine-rich protein of 68.5 kDa that is typically secreted in a highly glycosylated 88 kDa form [12] and expressed ubiquitously throughout the body [13]. In the central nervous system (CNS), it is detected in neurons and microglia, but in astrocytes and ependymal cells, little or no PGRN is detected [14–16]. It is reported that PGRN performs various biological functions, such as the regulation of cell growth, embryonic development, tissue repair, and the modulation of inflammation [17, 18]. Recently, the anti-inflammatory effects of PGRN in various states were reported, including acute lung injury, neurodegenerative diseases and ischemic stroke [19–21]. PGRN has been reported to have anti-inflammatory properties that may prevent brain injury by reducing the release of pro-inflammatory cytokines or elevating the release of anti-inflammatory factors [22]. In addition, PGRN-deficient mice displayed exaggerated inflammation compared with wild types [23].

However, until now, no study has focused on the anti-inflammatory effects of PGRN in SAH. In the present study, we investigated the time course of PGRN levels in cerebrospinal fluid (CSF) of SAH patients. In addition, the time course and anti-inflammatory effects of PGRN were explored in the brains of rats after experimental SAH.

## Methods

### Ethics statement

All procedures in the human study were approved by Jinling Hospital’s medical institutional review board and were all performed in accordance with the Declaration of Helsinki.

All experimental protocols including animal use and surgical procedures were approved by the Animal Care and Use Committee of Jinling Hospital and conformed to the Guide for the Care and Use of Laboratory Animals by the National Institutes of Health.

### Human CSF preparation

CSF was obtained from 43 SAH patients and 4 joint replacement patients (control group). The patients were divided into a control group (*n* = 4) and three SAH groups based on the time after SAH: 1–3 days (*n* = 16), 4–7 days (*n* = 18), and ≥8 days (*n* = 9). The injury severity scores including Hunt&Hess grade, Modified Fisher grades, World Federation of Neurological Surgeons (WFNS) Grading System, and Glasgow Coma Scale (GCS) were immediately recorded for SAH patients after admission to the hospital. The CSF was obtained through lumbar puncture and during surgery. After centrifugation (3000 g, 5 min), the supernatant of CSF was collected and stored at −80 °C. The clinical outcomes of these patients were evaluated at discharge according to Glasgow Outcome Scale (GOS) and Modified Rankin Scale (MRS). Detailed information was presented in Table 1.

**Table 1:** Clinical data from control and SAH cases

Case no	Age (year)/sex	Aneurysm location	Time after SAH (day)	Hunt&Hess grade	Fisher grade	WFNS	GCS	GOS	MRS
1	57/F	ACOA	1	3	4	2	13	3	3
2	57/F	ACOA	5	3	4	2	13	3	3
3	69/M	LACA	5	4	–	5	3	2	5
4	57/F	ACOA	6	3	4	2	13	3	3
5	69/M	LACA	6	4	–	5	3	2	5
6	57/F	ACOA	7	3	4	2	13	3	3
7	69/M	LACA	11	4	–	5	3	2	5
8	50/F	LMCA	3	2	2	1	15	3	4
9	57/F	ACOA	14	3	4	2	13	3	3
10	57/F	ACOA	15	3	4	2	13	3	3
11	63/M	ACOA	4	5	4	5	3	2	5
12	67/F	LICA	5	4	4	5	4	5	1
13	68/F	RPCOA	6	3	2	2	14	5	1
14	52/M	LMCA	1	4	4	4	7	3	5
15	54/M	LMCA	8	2	2	1	15	1	–
16	51/F	ACOA	15	2	3	2	7	3	4
17	58/F	ACOA	2	1	2	1	15	5	0
18	51/F	LICA	3	3	3	4	10	5	1
19	67/F	RPCOA	3	2	3	1	15	1	–
20	66/F	ACOA	2	1	3	3	15	5	1
21	67/F	RPCOA	6	2	3	1	15	1	–
22	62/F	ACOA	5	2	3	1	15	3	4
23	65/F	ACOA	5	2	2	1	15	5	0
24	58/M	ACOA	3	4	4	4	7	3	4
25	59/M	RMCA	4	2	2	1	15	5	1
26	59/M	RICA	4	5	4	5	3	2	5
27	43/F	LMCA	4	2	2	1	15	5	0
28	66/F	LPCOA	2	2	2	1	15	5	0
29	67/F	ACOA	1	2	2	2	14	4	2
30	59/M	RICA	20	5	4	5	3	2	5
31	67/F	ACOA	3	2	2	2	14	4	2
32	45/M	ACOA	10	2	2	1	15	3	4
33	46/F	ACOA	1	2	2	1	15	5	0
34	48/M	ACOA	2	2	2	1	15	5	0
35	59/M	ACOA	6	4	4	5	5	3	4
36	50/F	RMCA	2	3	2	2	13	3	4
37	59/M	ACOA	14	4	4	5	5	3	4
38	38/F	BT	1	3	4	4	8	4	2
39	38/F	BT	4	3	4	4	8	4	2
40	59/M	ACOA	1	5	4	5	3	3	4
41	50/F	RMCA	6	3	2	2	13	3	4
42	38/F	BT	10	3	4	4	8	4	2
43	69/M	LACA	4	4	–	5	3	2	5
44	58/M	–	–	–	–	–	–	–	–
45	55/F	–	–	–	–	–	–	–	–
46	49/F	–	–	–	–	–	–	–	–
47	52/M	–	–	–	–	–	–	–	–

*ACA* anterior cerebral artery, *ACOA* anterior communicating artery, *ICA* internal carotid artery, *MCA* middle cerebral artery, *PCOA* posterior communicating artery, *BT* basilar artery, *WFNS* World Federation of Neurological Surgeons Grading System, *GCS* Glasgow Coma Score, *GOS* Glasgow Outcome Scale, *MRS* Modified Rankin Scale

### Animal preparation and experiment design

Male Sprague–Dawley (SD) rats (280–320 g) were purchased from the Animal Center of Jinling Hospital, Jiangsu, China. The rats were raised under a 12-h light/dark cycle (25 ± 1 °C) and had free access to food and water at the Animal Center of Jinling Hospital.

Rats were randomly divided into six groups: sham, 12 h, and 1, 3, 5, and 7 days SAH groups, respectively. Rats were sacrificed for sample collection at the corresponding time points after SAH.

Based on PGRN results, we increased the levels of PGRN and sacrificed all animals at 24 h after SAH. Recombinant human PGRN (r-PGRN) (R&D Systems, Inc., Minneapolis, MN, USA) (r-PGRN in 5 μL of phosphate-buffered saline (PBS)) or an equal volume of PBS were administrated at 30 min after SAH through a single intracerebroventricular (i.c.v.) injection [24]. Animals were randomly divided into seven groups: sham, SAH, SAH + PBS, and SAH + PGRN (1, 3, 10, and 15 ng per rat). Initially, four concentrations were used to test the neuroprotective effects of r-PGRN on EBI after SAH. We chose these doses based on an ischemic stroke study [19]. Neurologic scores and brain water content were measured at 24 h after SAH. In the second set of experiments, which were based on our initial study, rats were treated with a dose of r-PGRN that gave the most marked effect for further studies. Six rats were used in each group.

### Animal model of SAH

**Fig. 1:**
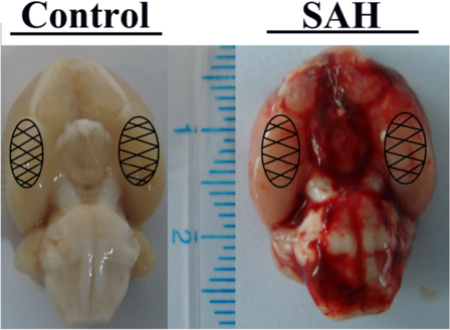
Experimental SAH model of rats. Schematic diagram of the areas used for assays. (Control) rat brain from the control group; (SAH) rat brain from the SAH group

Experimental SAH models were produced as reported previously [25]. In brief, the rats were anesthetized with chloral hydrate (0.4 mg/kg, IP, Jinling Hospital). The head and inguinal region was carefully shaved and disinfected. Rats were then fixed in a stereotaxic apparatus. A midline scalp incision was made and a 1-mm hole was drilled 8.0 mm anterior to Bregma in the midline. To prevent loss of CSF and bleeding from the midline vessels, we used bone wax to plug the burr hole. Rats were placed in the supine position and an insulin syringe (BD Science) was used to draw 300 μL of nonheparinized blood from the femoral artery. The needle was advanced 11 mm into the prechiasmatic cistern through the burr hole, at a 45° angle to the vertical plane, and then 300 μL of blood was injected into the prechiasmatic cistern over 20 s. An equal volume of normal saline was injected into the prechiasmatic cistern of rats in the sham group (Fig. 1). The burr hole was sealed with bone wax, and the incision was surgically sutured. Rats were kept at 30 °C, with their head placed in a downward position for 20 min. After recovery from anesthesia, the rats were returned to their cages and housed at 25 ± 1 °C. Rats that died during surgery or surgical recovery were excluded from the study, and the procedure was repeated until the final group size reached the planned experimental number.

The tail artery was cannulated to measure blood pressure (BP). For intracranial pressure (ICP) monitoring, a butterfly needle was percutaneously placed into cisterna magna through foramen magnum. And then the needle was connected to the tube of ICP monitoring device and transferred the pressure to the transducer [25, 26]. BP and ICP were recorded during blood injection and before the animals were killed.

### Neurologic scoring

We used three behavioral activity examinations (Table 2) to evaluate the neurologic function of rats at 24 h after SAH as previously described [27–29]. Grading of neurologic deficits was as follows: severe neurologic deficit (scores = 4–6), moderate neurologic deficit (scores = 2–3), mild neurologic deficit (scores = 1), and no neurologic deficit (scores = 0).

**Table 2:** Behavior scores

Category	Behavior	Score
Appetite	Finished meal	0
	Left meal unfinished	1
	Scarcely ate	2
Activity	Active, squeaking, or standing	0
	Lying down, will stand, and walk with some stimulation	1
	Almost always lying down	2
Deficits	No deficits	0
	Unable to walk because of ataxia or paresis	1
	Impossible to walk and stand because of ataxia and paresis	2

### Brain water content

Rat brains were removed at 24 h after SAH. The brains were weighed immediately to determine the wet weight and dried ar 80 °C for 72 h to determine the dry weight. The percentage of brain water content was calculated as follows: brain water content (percentage) = ((wet weight − dry weight) / wet weight) × 100 %.

### BBB permeability

We used Evans blue (EB) extravasation to assess BBB permeability at 24 h after SAH. As described previously [30], EB dye (2 %, 4 ml/kg) was injected intraperitoneally with a 3-h circulation time. Rats were anesthetized and perfused transcardially with 0.9 % normal saline solution (4 °C) to remove intravascular EB dye. Next, brains were removed and weighed. Brain samples were homogenized in physiological PBS (PH = 7.4) and centrifuged (15,000 g, 30 min). The resulting supernatant (0.5 mL) was added to an equal volume of 50 % trichloroacetic acid. After incubation overnight and centrifugation (15,000 g, 30 min, 4 °C), the supernatant was measured at 610 nm for spectrophotometric quantification. EB content was calculated as microgram per gram of protein.

### Perfusion—fixation and tissue preparation

Brains were harvested from rats after intracardiac perfusion with 0.9 % normal saline solution (4 °C) under anesthesia at 24 h after SAH. Brains that had obvious clots in the prechiasmatic cistern were selected for further analysis. After blood clots on the brain tissue were cleared, the temporal lobe tissue was harvested on ice and stored in −80 °C for protein extraction. For immunohistochemistry (IHC) and terminal deoxynucleotidyl transferase-mediated uridine 5’-triphosphate-biotin nick end-labeling (TUNEL), rats were perfused with 4 % buffered paraformaldehyde (4 °C) after 0.9 % normal saline solution (4 °C) perfusion, followed by immersion in 4 % buffered paraformaldehyde (4 °C).

### Protein extraction and western blotting analysis

To extract total protein, proper size of tissue was mechanically homogenized in 20 mM Tris (PH 7.6, 0.2 % SDS, 1 % Triton X-100, 1 % deoxycholate, 1 mM phenylmethylsulfonyl fluoride (PMSF), and 0.11 IU/ml aprotinin) (all from Sigma, St.Louis, MO, USA). Homogenate was centrifuged at 12,000 g for 15 min at 4 °C. The supernatant was collected and stored at −80 °C.

Equal amounts of protein were loaded in each lane of sodium dodecyl sulfate polyacrylamide gel electrophoresis (SDS-PAGE) and transferred to a polyvinylidene-difluoride (PVDF) membrane. The membrane was blocked for nonspecific binding with 5 % defatted milk for 2 h at room temperature and incubated overnight at 4 °C with primary antibodies. For primary antibodies, we used goat anti-PGRN (1:200; sc-11342, Santa Cruz, CA, USA), rabbit anti-Myeloperoxidase (MPO) (1:200; sc-16128, Santa Cruz, CA, USA), rabbit anti-zonula occludens 1 (ZO-1) (1:200; sc-10804, Santa Cruz, CA, USA), rabbit anti-matrix metalloproteinase-9 (MMP-9) (1:5,000; ab38898, Abcam), rabbit anti-bcl-2 (1:200; sc-492, Santa Cruz, CA, USA), rabbit anti-cleaved caspase-3 (1:5,000; 9661, Cell Signaling, Beverly, MA, USA), and rabbit anti-β-actin (1:5,000; AP0060, Bioworld Technology, Minneapolis, MN, USA). After being washed with TBST (3 × 10 min), the membrane was incubated with rabbit anti-goat or goat anti-rabbit horseradish peroxidase (HRP)-conjugated IgG (1:5,000, BS30503 and BS13278, Bioworld Technology, Minneapolis, MN, USA) for 2 h at room temperature. Using the enhanced chemiluminescence reagent kit (Millipore Corporation, Billerica, MA, USA), bands were visualized. Quantification was performed by optical density methods using ImageJ software (NIH), and the data was normalized to β-actin.

### Enzyme-linked immunosorbent assay

Total protein was determined using a bicinchoninic acid assay kit (Pierce Biochemicals). The inflammatory cytokine levels of brain tissue were quantified using enzyme-linked immunosorbent assay (ELISA) kits specific for rats according to the manufacturer’s instructions (PGRN, P28799, from Raybiotech, USA; MPO, ab119605, from Abcam, USA; tumor necrosis factor-α (TNF-α), 950.090.096 and 865.000.096, from Diaclone Research, France; IL-1β, 850.006.096 and 670.040.096, from Diaclone Research, France). The detailed method is shown as follows: wash microwell strips twice with wash buffer. Standard dilution on the microwell plate is as follows: add 100 μl sample diluents, in duplicate, to all standard wells. Pipette 100 μl prepared standard into the first wells and create standard dilutions by transferring 100 μl from well to well. Discard 100 μl from the last wells. Alternatively, external standard dilution in tubes is as follows: pipette 100 μl of these standard dilutions in the microwell strips. Add 100 μl sample diluents, in duplicate, to the blank wells. Add 50 μl sample diluents to sample wells. Add 50 μl sample in duplicate, to designate sample wells. Cover microwell strips and incubate 2 h at room temperature. Empty and wash microwell strips five times with wash buffer. Add 100 μl Biotin—conjugate to all wells. Cover microwell strips and incubate 1 h at room temperature. Empty and wash microwell strips five times with wash buffer. Add 100 μl diluted streptavidin—HRP to all wells. Cover microwell strips and incubate 1 h at room temperature. Empty and wash microwell strips five times with wash buffer. Add 100 μl of TMB substrate solution to all wells. Incubate the microwell strips for about 10 min at room temperature. Add 100 μl stop solution to all wells. Blank microwell reader and measure color intensity at 450 nm. Inflammatory cytokine levels in the CSF of patients were calculated as pictogram per milliliter, and inflammatory cytokine levels in the brain cortex of rats were calculated as pictogram per milligram.

### IHC and TUNEL

IHC was performed as previously described [31]. Rat brains, which had been fixed with 4 % buffered paraformaldehyde, were embedded in paraffin and cut into 10-μm slices. The sections were deparaffinized and incubated with 3 % H_2_O_2_ in PBS for 10 min. The sections were blocked with 5 % normal fetal bovine serum in PBS for 2 h followed by incubation with primary antibodies overnight at 4 °C. For primary antibodies, we used goat anti-PGRN antibody (1:200; sc-11342, Santa Cruz, CA, USA) and rabbit anti-MPO antibody (1:200; sc-16128, Santa Cruz, CA, USA). Each section was then incubated with rabbit anti-goat or goat anti-rabbit horseradish peroxidase (HRP)-conjugated IgG (1:500, BS30503 and BS13278, Bioworld Technology, Minneapolis, MN, USA) at room temperature for 60 min. Diaminobenzidine (DAB) and counterstaining were performed with hematoxylin. Positive cells were identified, counted, and analyzed under the light microscope by an investigator blinded to the groupings. Six random high power fields (400×) in each coronary section were selected, and the mean percentage of positive cells in the six fields was used for final analysis. A total of 10 sections from each sample were used for quantification.

According to our previous study [28], TUNEL staining was performed using an in situ cell death detection kit (Roche, Indianapolis, IN, USA). The severity of brain damage was evaluated by the apoptotic index, which was defined as the average percentage of TUNEL-positive cells. The TUNEL-positive cells were identified, counted, and analyzed under the light microscope by an investigator blinded to the groupings. Six random high power fields (400×) in each coronary section were selected, and the mean percentage of apoptotic neurons in the six fields was used for final analysis. A total of 10 sections from each sample were used for quantification.

### Statistical analysis

All data that were used for the statistical analysis was presented as mean ± SEM (SPSS 17.0 and GrahPad Prism 5.0 software). Mortality rate between groups was analyzed by Fischer exact test. Other data was subjected to a one-way analysis of variance followed by Tukey post hoc test. The level of statistical significance was inferred at *p* < 0.05.

## Results

### Levels of PGRN decreased in the CSF of SAH patients

**Fig. 2:**
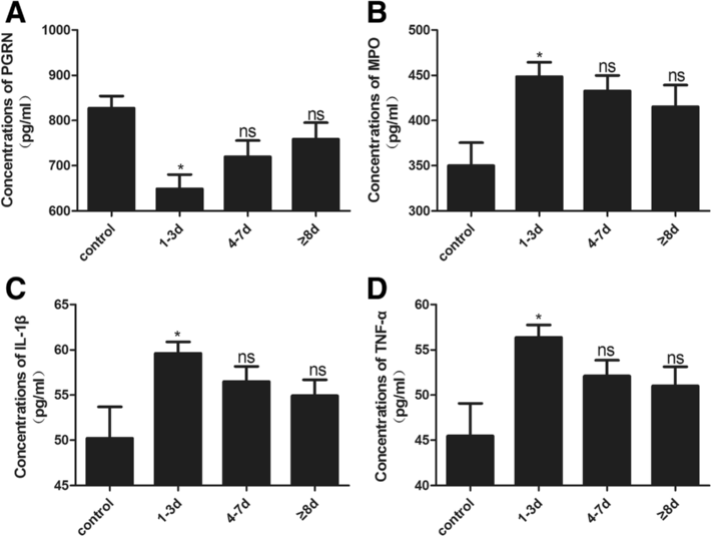
ELISA analysis of PGRN, MPO, IL-β, and TNF-α in the CSF of patients. The concentration of PGRN decreased after SAH and was lowest in the 1–3 days group (**a**). However, the concentration of MPO, IL-β, and TNF-α increased after SAH and was highest in the 1–3 days group (**b**–**d**). Data are expressed as the mean ± SEM from six rats. *p < 0.05 compared with the control group, ^ns^ *p* > 0.05 compared with the control group

PGRN levels as assessed by ELISA were summarized in Fig. 2a. High levels of PGRN were detected in the control group, while decreased levels were observed in the CSF of SAH patients (1–3 days, *p* < 0.05; 4–7 days and ≥8 days, *p* > 0.05). In addition, the levels of MPO, IL-1β and TNF-α were low in the control group and increased in the SAH groups (1–3 days, *p* < 0.05; 4–7 days and ≥8 days, *p* > 0.05) (Fig. 2b–d). The results showed that PGRN levels decreased after SAH, which may be associated with the levels of MPO, IL-1β, and TNF-α.

### General observations and mortality rate

After SAH, no statistical difference in physiological parameters (BP and ICP) was observed among the groups (data not shown). There were 221 rats used in the present study and the mortality rates during and after surgery were as follows: (1) the sham group (0 of 6 rats), the SAH groups on 12 h (1 of 7 rats), 1 days (2 of 8 rats), 3 days (1 of 7 rats), 5 days (2 of 8 rats), and 7 days (2 of 8 rats); (2) the sham group (0 of 30 rats), the SAH group (6 of 36 rats), the SAH + PBS group (5 of 35 rats), the SAH + PGRN (1 ng per rat) (2 of 14 rats), the SAH + PGRN (3 ng per rat) (3 of 13 rats), the SAH + PGRN (10 ng per rat) (5 of 35 rats), and the SAH + PGRN (15 ng per rat) (2 of 14 rats).

### Temporal alterations of PGRN protein levels after experimental SAH in rats

**Fig. 3:**
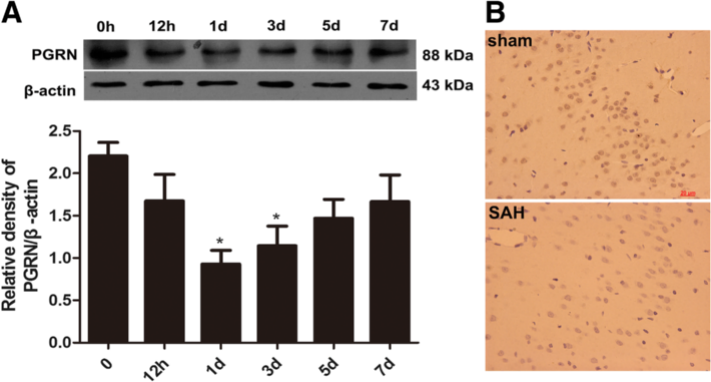
Western blot analysis and IHC of PGRN in the brain cortex of rats. PGRN protein levels decreased after SAH and were lowest at 24 h after SAH (**a**). IHC revealed that there were more PGRN-positive cells in the sham group compared with the 24-h SAH group (**b**). Data are expressed as the mean ± SEM from six rats. *p < 0.05 compared with the control group

The time course of PGRN protein levels were assessed by western blotting. PGRN protein levels decreased at 12 h after SAH with the lowest level observed at 24 h (Fig. 3a). According to the western blotting results, we selected the sham group and the 24-h group (lowest level of PGRN) for IHC. PGRN-positive cells markedly decreased at 24 h after SAH compared with the sham group (Fig. 3b).

### R-PGRN significantly improves neurologic function and reduces brain water content at 24 h after SAH

**Fig. 4:**
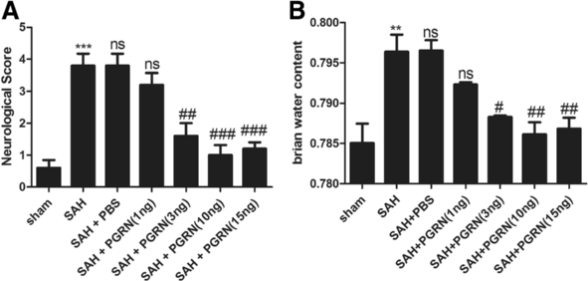
Effects of PGRN on neurologic scores (**a**) and brain water content (**b**) at 24 h after SAH. Neurological function of rats was impaired and brain water content increased significantly at 24 h after SAH compared with rats in the sham group. R-PGRN (3, 10, and 15 ng/rat) dramatically increased neurological scores and alleviated brain edema at 24 h after SAH, but r-PGRN at a dose of 1 ng/rat did not significantly affect neurological scores and brain edema. No difference was detected between the untreated SAH group and the PBS treated SAH group. Data are expressed as the mean ± SEM from six rats. ***p < 0.001 and **p < 0.01 compared with the sham group, ^ns^ *p* > 0.05 compared with the SAH group, ^###^ *p* < 0.01, ^##^ *p* < 0.01, and ^#^ *p* < 0.05 compared with the SAH group

To test the neuroprotective effects of r-PGRN on EBI, we evaluated the neurologic function and brain water content at 24 h after SAH. In this study, r-PGRN was given at four different concentrations to SAH rats. As shown in Fig. 4, treatment with r-PGRN at 3, 10, and 15 ng/rat dramatically ameliorated neurologic function (*p* < 0.01, *p* < 0.001) and brain edema (*p* < 0.05, *p <* 0.01). However, r-PGRN at 1 ng/rat had no significant effect. In addition, r-PGRN at a higher dose of 15 ng/rat did not exhibit a better neuroprotective effect than that at 10 ng/rat. These results suggested that r-PGRN could alleviate EBI and that r-PGRN at 10 ng/rat exhibited the most beneficial effect. Therefore, we selected 10 ng/rat for further experiments.

### PGRN suppresses the recruitment of neutrophils at 24 h after experimental SAH in rats

**Fig. 5:**
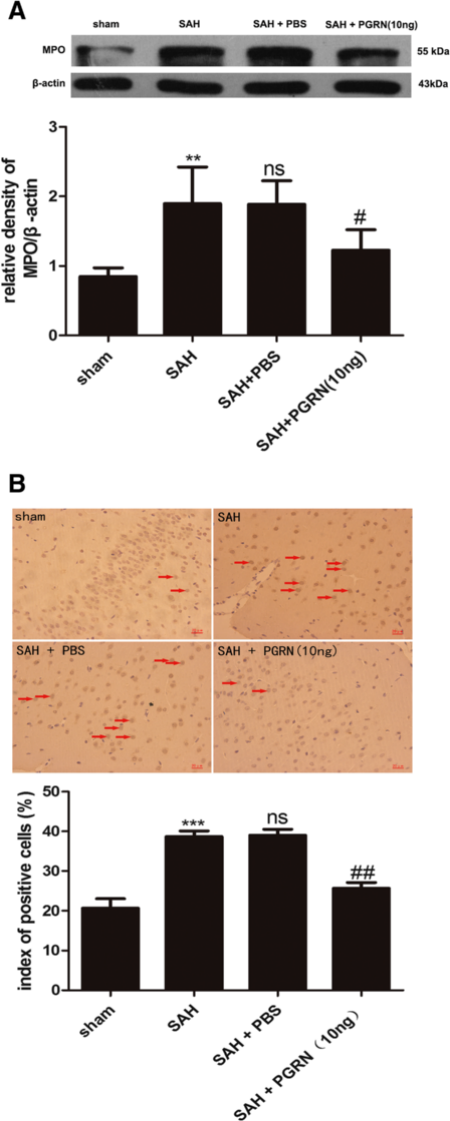
Western blot analysis and IHC of MPO in the brain cortex of rats 24 h after SAH. MPO protein levels increased after SAH and significantly decreased following r-PGRN treatment (**a**). IHC revealed that MPO-positive cells decreased after treatment with PGRN, which significantly increased after SAH (**b**). There was no difference between the untreated SAH group and the PBS-treated SAH group. Data are expressed as the mean ± SEM from six rats. ***p < 0.001 and **p < 0.01 compared with the sham group, ^ns^ *p* > 0.05 compared with the SAH group, ^##^ *p* < 0.01 and ^#^ *p* < 0.05 compared with the SAH group

Western blotting (Fig. 5a) showed that the protein levels of MPO significantly increased in the SAH and SAH + PBS groups (*p <* 0.01) and decreased in the SAH + PGRN group (*p* < 0.05). IHC (Fig. 5b) revealed that MPO-positive cells in the SAH and SAH + PBS groups were significantly increased relative to that of the sham group (*p* < 0.001). Following r-PGRN treatment, the numbers of MPO-positive cells decreased (*p <* 0.01).

### R-PGRN reduces the production of pro-inflammatory cytokines at 24 h after experimental SAH in rats

**Fig. 6:**
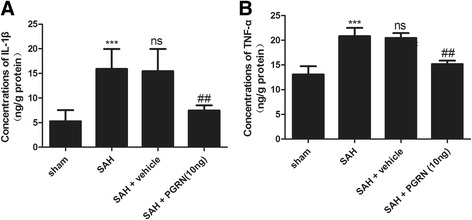
ELISA analysis of IL-β and TNF-α in the brain cortex of rats 24 h after SAH. The concentrations of  IL-β (**a**) and TNF-α (**b**) significantly increased at 24 h after SAH and decreased after administration of r-PGRN. No difference was detected between the untreated SAH group and the PBS-treated SAH group. Data are expressed as the mean ± SEM from six rats. ***p < 0.001 compared with the sham group, ^ns^ *p* > 0.05 compared with the SAH group, ^##^ *p* < 0.01 compared with the SAH group

ELISA was used to measure the production of pro-inflammatory cytokines (TNF-α and IL-1β). As shown in Fig. 6, the concentrations of cytokines were significantly increased at 24 h after SAH (*p* < 0.001) and significantly reduced after treatment with r-PGRN (*p <* 0.01).

### Brain edema is ameliorated with r-PGRN treatment at 24 h after experimental SAH in rats

**Fig. 7:**
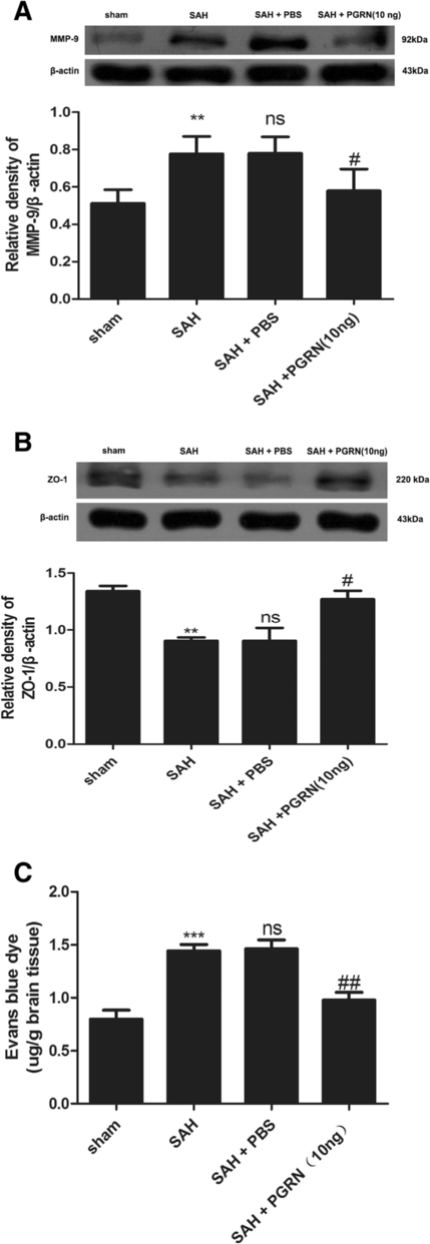
The effect of PGRN on MMP-9 and ZO-1 levels in the brain cortex of rats and extravasation of EB dye at 24 h after SAH. Western blotting revealed that MMP-9 levels in the SAH group were significantly higher than that in the sham group. R-PGRN treatment reduced the levels of MMP-9 (**a**). ZO-1 protein levels in the SAH group were significantly lower than that in the sham group. Treatment with r-PGRN markedly increased ZO-1 levels (**b**). EB showed significantly higher BBB permeability in the SAH group compared with the sham group. R-PGRN administration significantly reduced BBB permeability induced by SAH (**c**). There was no difference between the untreated SAH group and the PBS-treated SAH group. Data are expressed as the mean ± SEM from six rats. ***p < 0.001 and **p < 0.01 compared with the sham group, ^ns^ *p* > 0.05 compared with the SAH group, ^##^ *p* < 0.01 and ^#^ *p* < 0.05 compared with the SAH group

To demonstrate the effects of PGRN on brain edema, BBB permeability and protein levels of MMP-9 and ZO-1 were tested at 24 h after SAH. MMP-9 levels and ZO-1 degradation significantly increased in the SAH and SAH + PBS groups (*p <* 0.01; Fig. 7a, b) and were largely reduced after r-PGRN treatment (*p* < 0.05; Fig. 7a, b). As shown in Fig. 7c, EB indicated that BBB permeability increased after SAH (*p* < 0.001) and dramatically reduced after PGRN treatment (*p <* 0.01).

### Increased PGRN decreases neural cell apoptosis at 24 h after experimental SAH in rats

**Fig. 8:**
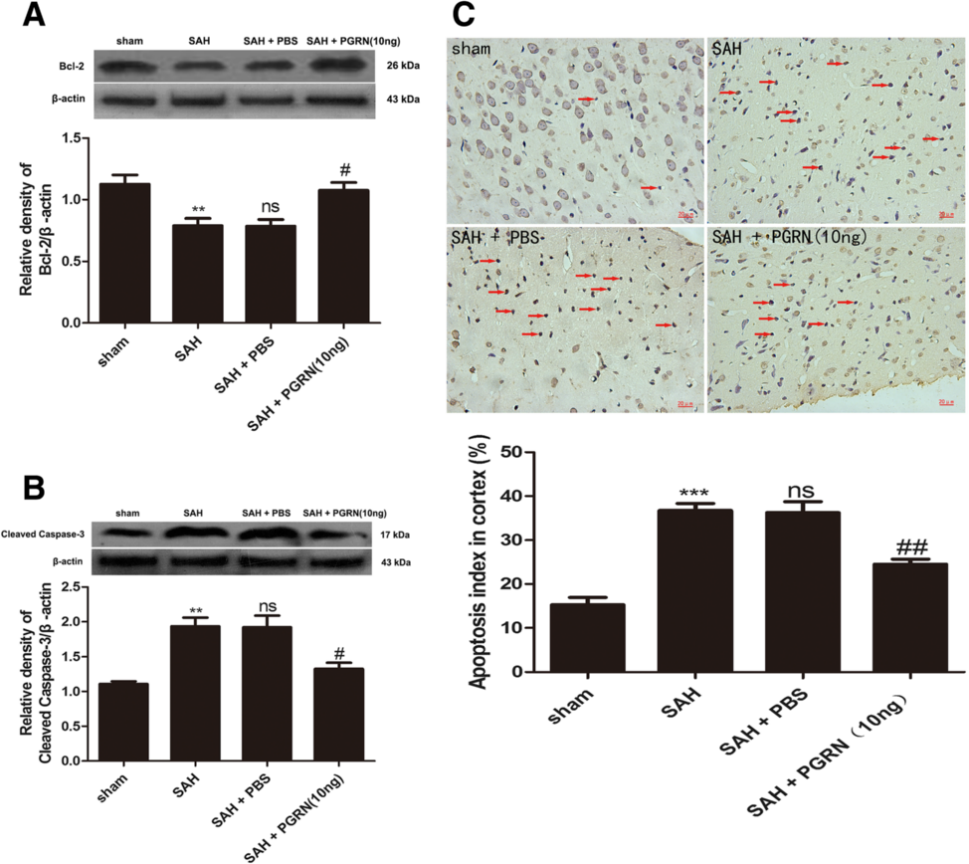
Effect of PGRN on neural cell apoptosis 24 h after SAH. Western blotting revealed Bcl-2 levels in the SAH group were significantly reduced compared with sham. R-PGRN treatment increased the levels of Bcl-2 (**a**). Cleaved caspase-3 levels in the SAH group were significantly higher than the sham group. Treatment with r-PGRN markedly reduced cleaved caspase-3 levels (**b**). The number of TUNEL-positive cells increased after SAH and reduced after r-PGRN treatment (**c**). No difference was detected between the untreated SAH group and the PBS-treated SAH group. Data are expressed as the mean ± SEM from six rats. ***p < 0.001 and **p < 0.01 compared with the sham group, ^ns^ *p* > 0.05 compared with the SAH group, ^##^ *p* < 0.01 and ^#^ *p* < 0.05 compared with the SAH group

To illustrate the effects of PGRN on neural cell apoptosis, the levels of apoptosis-related proteins (bcl-2 and cleaved caspase-3) and extent of TUNEL were monitored at 24 h after SAH in our study. As shown in Fig. 8a, b, Bcl-2 levels decreased after SAH (*p <* 0.01) and were upregulated by r-PGRN treatment (*p* < 0.05). In contrast, cleaved caspase-3 levels increased after SAH (*p <* 0.01) and reduced after treatment with r-PGRN (*p* < 0.05). In addition, TUNEL (Fig. 8c) revealed that there were few TUNEL-positive cells in the sham group, whereas the apoptotic index in the SAH group was significantly higher than that of rats in the sham group (*p* < 0.001). The percentage of TUNEL-positive cells reduced after r-PGRN treatment (*p <* 0.01).

## Discussion

In the present study, we demonstrated for the first time that PGRN decreased in the CSF of SAH patients and reached a maximum low at 1–3 days after SAH. In addition, the decrease of PGRN was associated with an increase in MPO, IL-1β, and TNF-α. To clarify the role of PGRN following SAH, we continued our research in experimental rats. PGRN decreased in the brain of rats after SAH with a maximum low at day 1. Furthermore, we found that PGRN treatment alleviated EBI by suppressing neutrophil recruitment. Our results suggested that increased PGRN was involved in inhibiting the neutrophil-mediated inflammatory response after SAH.

EBI has been demonstrated as a primary cause of high morbidity and mortality [1]. Therefore, there is a need to devise therapeutic strategies for improving outcomes of SAH patients [5]. A large body of evidence demonstrates that inflammation appears to be the proegumenal cause of brain injury after SAH [32] and that patients with high levels of cytokines (such as IL-1β and TNF-α) would have unfavorable outcomes and symptoms for clinical deterioration [33]. Inflammation induces apoptosis [34] and leads to BBB disruption by increasing endothelial permeability and vessel diameter [35]. In addition, a number of studies have indicated the critical role of neutrophils in the inflammatory response [36]. Neutrophils are among the first cells in the blood to respond after brain injury and are a main cause of brain injury [11, 37]. Neutrophils release pro-inflammatory mediators and chemotactic agents, enhancing the recruitment of further neutrophils and exacerbating inflammation [38]. Activated neutrophils can modulate the level of MMP-9 [39], which is reported to participate in BBB damage and brain edema by degrading tight junction proteins and basal lamina proteins after SAH [40, 41]. Previous research has shown that limiting neutrophil activity reduces vascular collagenase activity and microvessel wall injury and increases in vessel permeability, all of which alleviates brain injury after SAH [42].

Previous studies have demonstrated the anti-inflammatory role of PGRN in CNS diseases, such as neurodegenerative diseases and ischemic stroke [19, 43], and that deficiency of PGRN resulted in exaggerated inflammation and BBB disruption [13]. Moreover, PGRN modulates neutrophilic inflammation by inhibiting the activation and recruitment. However, activated neutrophils can secrete neutrophil elastase (NE) to degrade PGRN into individual granulin peptides, which act in the opposite manner, stimulating the production of pro-inflammatory cytokines [17, 34]. Therefore, we hypothesized that a decrease in PGRN levels potentiates the inflammation induced by EBI and that high levels of PGRN may inhibit inflammation by suppressing neutrophil activation. In our study, we found that PGRN levels decreased in the CSF of SAH patients. In addition, changes in PGRN levels occurred in a time-dependent manner, in contrast to MPO, IL-1β, and TNF-α levels. Based on these results, we suggest that PGRN can alleviate EBI and that this protective role is related to inflammation. This hypothesis is supported in our animal study. We demonstrated that PGRN decreased after SAH in the brain of rats and that increased PGRN could alleviate EBI. We found r-PGRN treatment could suppress neutrophil recruitment and reduce the production of pro-inflammatory cytokines such as TNF-α and IL-1β. In addition, we demonstrated that r-PGRN alleviated EBI after SAH. Our results were also in line with Friedrich et al.’s findings [38] and confirmed that neutrophils contributed to earlier inflammation after SAH. Furthermore, present results show that reduction in PGRN is one of the important contributors of neutrophil-induced inflammation.

Brain edema is a key factor related to the mortality and poor outcome after SAH [44]. Moreover, it is associated with BBB breakdown caused by disassembly of tight junctions and endothelial cell contraction [45]. A previous study indicates that PGRN deficiency leads to major alterations in BBB structure and function [13]. In our study, we found that r-PGRN administration reduced the levels of MMP-9 and concomitantly restored the levels of ZO-1, which is one of the tight junction proteins. Meanwhile, BBB permeability, as assessed by extravasation of EB dye in the brain, was largely reduced by r-PGRN. These findings demonstrated that PGRN protected the BBB by blocking the increase of MMP-9 and the degradation of tight junction proteins after SAH.

Previous studies have shown that apoptosis is another contributor to a poor outcome after SAH [46] and can be reduced by PGRN [47]. Our current study observed that PGRN decreased the levels of apoptosis-related proteins (Bcl-2 and cleaved caspase-3). Moreover, TUNEL demonstrated that PGRN could reduce neuronal apoptosis after SAH.

Accumulating evidence has demonstrated the protective role of PGRN in brain injury, and a recent study showed that PGRN could reduce neuronal cell death after SAH [48]. However, the anti-inflammatory role of PGRN after SAH is still unknown. Our study indicated an anti-inflammatory role of PGRN on EBI after SAH by inhibiting the recruitment and activation of neutrophils, which is emerging as a hallmark of vascular inflammation [9] and is closely related to BBB breakdown [49]. Based on previous studies and our study, the protective role of PGRN on EBI has been identified [48]. However, further studies are needed to validate the exact role and mechanism of PGRN after SAH.

Although our study has some limitations, such as the lack of different severities of SAH and the short-term follow-up in patients, we demonstrated that PGRN could reduce neuronal death, alleviate brain edema, preserve the BBB, and inhibit inflammation by suppressing neutrophil recruitment. However, these effects may act via different pathways, such as the sortilin and nuclear factor-κB signal pathway. Hence, further studies are needed to explain the full role of PGRN in the pathophysiology of SAH.

## Conclusions

In conclusion, our study demonstrated that the levels of PGRN decreased in the CSF from SAH patients and in the brain cortex of rats after SAH. In addition, an administration of r-PGRN alleviated EBI, which was associated with inhibiting the inflammatory reaction by suppressing neutrophil recruitment. Moreover, PGRN inhibited BBB disruption and reduced neural cell apoptosis, which are both important contributors to a poor outcome after SAH.

## Abbreviations

SAH: subarachnoid hemorrhage
EBI: early brain injury
PGRN: progranulin
r-PGRN: recombinant human PGRN
MPO: myeloperoxidase
IL-1β: interleukin1β
TNF-α: tumor necrosis factor-α
CSF: cerebrospinal fluid
ELISA: enzyme-linked immunosorbent assay
IHC: immunohistochemistry
PBS: phosphate buffered saline
MMP-9: matrix metalloproteinase-9
ZO-1: zonula occludens 1
BBB: blood brain barrier
CVS: cerebral vasospasm
CNS: central nervous system
WFNS: World Federation of Neurological Surgeons
GCS: Glasgow Coma Scale
GOS: Glasgow Outcome Scale
MRS: Modified Rankin Scale
SD: Sprague–Dawley
EB: Evans blue
TUNEL: terminal deoxynucleotidyl transferase-mediated uridine 5’-triphosphate-biotin nick end-labeling
PMSF: phenylmethylsulfonyl fluoride
